# Failure of Urological Implants in Spinal Cord Injury Patients due to Infection, Malfunction, and Implants Becoming Obsolete due to Medical Progress and Age-Related Changes in Human Body Making Implant Futile: Report of Three Cases

**DOI:** 10.1155/2013/826748

**Published:** 2013-06-20

**Authors:** Subramanian Vaidyanathan, Bakul Soni, Gurpreet Singh, Peter Hughes, Fahed Selmi, Paul Mansour

**Affiliations:** ^1^Regional Spinal Injuries Centre, Southport and Formby District General Hospital, Town Lane, Southport PR8 6PN, UK; ^2^Department of Urology, Southport and Formby District General Hospital, Town Lane, Southport PR8 6PN, UK; ^3^Department of Radiology, Southport and Formby District General Hospital, Town Lane, Southport PR8 6PN, UK; ^4^Department of Cellular Pathology, Southport and Formby District General Hospital, Town Lane, Southport PR8 6PN, UK

## Abstract

Any new clinical data, whether positive or negative, generated about a medical device should be published because health professionals should know which devices do not work, as well as those which do. We report three spinal cord injury patients in whom urological implants failed to work. In the first, paraplegic, patient, a sacral anterior root stimulator failed to produce erection, and a drug delivery system for intracavernosal administration of vasoactive drugs was therefore implanted; however, this implant never functioned (and, furthermore, such penile drug delivery systems to produce erection had effectively become obsolete following the advent of phosphodiesterase type 5 inhibitors). Subsequently, the sacral anterior root stimulator developed a malfunction and the patient therefore learned to perform self-catheterisation. In the second patient, also paraplegic, an artificial urinary sphincter was implanted but the patient developed a postoperative sacral pressure sore. Eight months later, a suprapubic cystostomy was performed as urethral catheterisation was very difficult. The pressure sore had not healed completely even after five years. In the third case, a sacral anterior root stimulator was implanted in a tetraplegic patient in whom, after five years, a penile sheath could not be fitted because of penile retraction. This patient was therefore established on urethral catheter drainage. Later, infection with *Staphylococcus aureus* around the receiver block necessitated its removal. In conclusion, spinal cord injury patients are at risk of developing pressure sores, wound infections, malfunction of implants, and the inability to use implants because of age-related changes, as well as running the risk of their implants becoming obsolete due to advances in medicine. Some surgical procedures such as dorsal rhizotomy are irreversible. Alternative treatments such as intermittent catheterisations may be less damaging than bladder stimulator in the long term.

## 1. Introduction

The Science and Technology Committee, House of Commons, United Kingdom, in its report on Regulation of Medical Implants in the European Union and UK, recommended the reporting of adverse incidents by healthcare professionals. “As there is some evidence of under reporting, the Government should make the reporting of adverse incidents mandatory for healthcare professionals.” Professor Westaby referred to the practice of selective publishing whereby positive results were reported and negative results were not. “It seemed, at present, surgeons were loath to admit problems in case they appeared to make them look incompetent.” The British Standards Institute agreed that *reporting by healthcare professionals should be mandatory* to “ensure that all reports are available to the appropriate medical device manufacturers so that manufacturers can fulfil their vigilance reporting and incident investigation obligations.” Under “Conclusions and Recommendations,” the Committee stated that any new clinical data generated about a medical implant should be published. “It is as scientifically useful to know what does work as it is to know what does not work.” [1].

We report three spinal cord injury patients in whom urological implants developed malfunctions, necessitating alternative modes of treatment. Apart from malfunctioning of implants, we also wish to highlight the following problems which spinal cord injury patients may encounter after undergoing surgery for urological implants.

1. Persons with spinal cord injury are at greater risk of developing complications such as pressure sores, chest infection, and wound infection than able-bodied individuals.
2. A spinal cord injury patient may find difficulty in using the implanted urological device after a number of years because of age-related changes in human anatomy and physiology. For example, with advancing age, a male spinal cord injury patient might develop abdominal obesity and retraction of penis, making the wearing of a penile sheath very difficult or even impossible. An implanted bladder stimulator often requires wearing a penile sheath and would therefore not be suitable for a spinal injury patient who is unable to wear a sheath securely, and who therefore would need bladder drainage by an indwelling urinary catheter instead. In hindsight, the patient (and indeed their spinal cord physician) might wonder whether alternative treatments should have been considered instead of the implant.
3. Implantation of a urological device often involves an irrevocable surgical procedure. For example, rhizotomy of sacral dorsal nerve roots is performed while implanting sacral anterior root stimulator, but is a permanent, irreversible procedure.
4. With medical progress, less invasive treatments may become available which effectively make the implant redundant, but removal would require a further surgical procedure. For example, implantable drug delivery systems for intracavernosal administration of vasoactive agents are no longer required as orally administered phosphodiesterase type 5 inhibitors have proved to be a simple and effective treatment for erectile dysfunction.

The following three case reports serve as examples of the above-mentioned shortcomings of implants in spinal cord injury patients.

## 2. Case Presentations

**Case 1**

**Figure 1:**
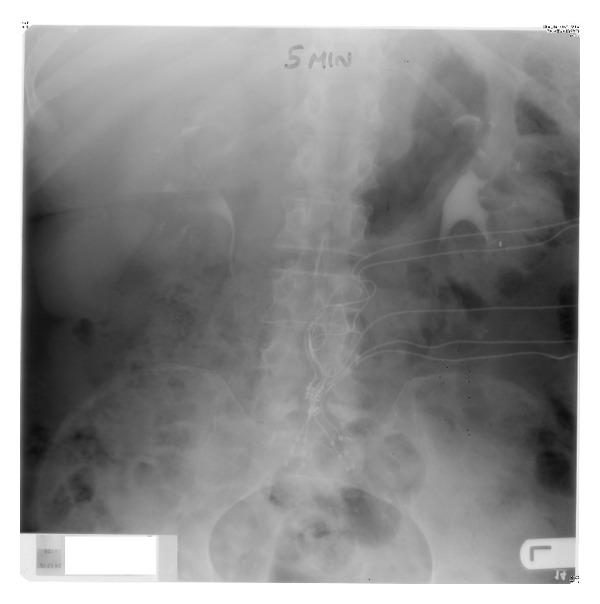
Case 1: five-minute film of intravenous urography, performed in December 2004, showing excretion of contrast by both kidneys with no hydronephrosis. The intradural and extradural electrodes of the sacral anterior nerve roots can be seen.

A 36-year-old Caucasian male sustained a T-8 complete paraplegia in a road traffic accident in 1981 and initially was managing his bladder with a penile sheath. In 1986, aerodynamics revealed very little rise in pressure when bladder was filled to 650 mL, followed by a contraction to 75 cm of H_2_O which expelled 250 mL, leaving a residual volume of 400 mL. The bladder was slightly trabeculated. That year, a sacral anterior root stimulator was implanted. In 1987, with the stimulus parameters that the patient was then using, he had a very large residual volume (750 mL), a high voiding pressure (up to 150 cm H_2_O) and manifest detrusor-sphincter dyssynergia. By adjusting the stimulus parameters, the residual volume of urine was decreased to 150 mL, but without any reduction in voiding pressure. Section of the posterior sacral nerve roots was therefore performed. Following deafferentation, urodynamics showed very high voiding pressures (150 cm H_2_O) and a urine flow rate of less than 5 mL per second. In June 1991, the bladder stimulator changed its behaviour, in a way that almost certainly indicated a fault in the S-2 channel. In August 1991, after surgical exposure of the implant confirmed the fault in S-2 and established that the fault was too close to the vertebral column to be repaired, new extradural electrodes were implanted and connected to a new receiver. Postoperatively, the S-2 electrodes worked, as stimulating S-2 produced bilateral plantar flexion but the S-2 channel ceased to work within a few days, again due to electrical failure of the new S-2 channel; stimulus of the S-2 channel gave feeble plantar flexion on the right side only. Sliding the transmitter about did not alter this, but pushing on the tunnel connector block reversibly altered the response to strong bilateral plantar flexion. This finding was consistent with what one would expect if there were a pair of wires in the S-2 channel separated by a saline bridge, but capable of being brought into direct contact by mechanical distortion. Despite using S-2/3/4 on a continual function, penile erection was not achieved. The patient was therefore taught self-injection of papaverine into corpora cavernosa, but developed massive bruising after a manual syringe. Subsequently, this patient was shown how to use the Mumford Autoinjector System, and a 15 mg injection of Papaverine produced an erection useful for intercourse. In January 1994, a Brindley Penile Drug Delivery System [2] was implanted through a midline penoscrotal incision as follows: the corpus cavernosum was dissected and the spongiocorporal junction was identified; corporotomy was performed and the delivery tube was implanted; a midline scrotal space was developed and the reservoir implanted. However, according to the patient, the pump never worked. In 2004, intravenous urography revealed excretion of contrast by both kidneys (Figure 1); there was no hydronephrosis and no radioopaque urinary calculus was seen. Ultrasound examination of urinary tract in 2012 revealed no hydronephrosis; a 5 mm calculus was noted in midpole of right kidney, and the outline of urinary bladder was normal. In 2013, the sacral root stimulator was not working as well as it should be, and the patient was performing self-catheterisation. The stimulator did not work for bowels either and he required manual evacuation of faeces.

**Case 2**

A 44-year-old Caucasian male sustained a gun shot injury to his back in 2004 and sustained paraplegia at L-2 in 2004. This patient was performing self-catheterisation and wearing a penile sheath and was taking oxybutynin 25 mg a day by mouth. However, he wanted to stop using the sheath. In 2007, video-urodynamics was performed while taking oxybutynin; detrusor pressure rose to about 50 cm H_2_O over a 480 mL fill. There was no vesicoureteric reflux and the bladder neck was wide open.

Detrusor myomectomy and implantation of the AMS Sphincter 800 Urinary Control System were carried out in 2008. Following surgery, the patient developed collapse of lower lobe of right lung for which he required chest physiotherapy and intermittent positive pressure ventilation. He also developed a large sacral pressure sore. The artificial sphincter was deactivated and the patient was advised to perform self-catheterisation. However, six months later he was unable to insert a size 10 French catheter for intermittent catheterisation and he was advised to use size 8 French catheters and to not allow his bladder to become overdistended. Subsequently, however, the patient still found difficulty in performing urethral catheterisations, describing the attempts as though the catheter was “hitting a brick wall,” there was no bleeding. Because of this, he would often go without catheterisation for 11 to 20 hours on a regular, almost daily, basis, causing the bladder to become distended with up to 1200 mL of urine. Therefore, suprapubic cystostomy was performed eight months after the original surgery; since then, this patient had no further urinary problems, although the sacral pressure sore had still not healed completely by 2013, five years later.

**Case 3**

In 1988, a 30-year-old Caucasian male dived into shallow water in a hotel swimming pool, sustaining a fracture of the fourth cervical vertebra and tetraplegia at C-4 level. His neuropathic bladder was initially managed by indwelling urethral catheter drainage. In 1990, the posterior roots of sacral 1, 2, 3, and 4 were cut and the anterior sacral roots 2, 3, and 4 were trapped in the bladder stimulator. Cables were tunnelled to the right flank and connected to the receiver block. In 1994, urodynamics revealed a detrusor pressure of 140 cm H_2_O on stimulation of the second anterior sacral nerve, 240 cm H_2_O while stimulating the third anterior sacral nerve, and 60 cm H_2_O when the fourth anterior sacral nerve was stimulated. In view of these very high detrusor pressures, the stimulus parameters were adjusted. In 1992, the stimulus parameters were found to produce powerful detrusor contractions, which resulted in the penile sheath coming off. In 1994, while using the sacral anterior nerve stimulator, the penis underwent retraction causing the penile sheath to come off, and an indwelling urethral catheter was therefore inserted. The stimulus parameters were adjusted and the programme for penile erection was increased in channel 1, but without effect; further adjustment still did not cause bladder emptying, and he therefore resumed indwelling urethral catheter drainage. In 1998, bladder biopsy showed nonspecific acute and chronic cystitis without ulceration. In 2005, urine cytology showed benign epithelial cells only with no red blood cells or significant inflammation and with no evidence of malignancy.

**Figure 2:**
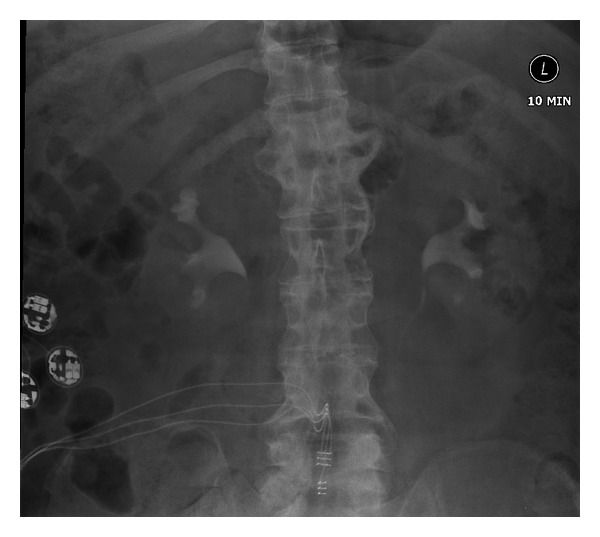
Case 2: ten-minute film of intravenous urography, performed in February 2008, showing some blunting of the right upper pole calyx with some reduction in cortical thickness at the right upper pole compatible with chronic pyelonephritis. Otherwise, both pelvicalyceal systems and ureters appear normal. The intradural electrodes of the sacral anterior nerve roots and the receiver block implanted in right flank can be seen.

**Figure 3:**
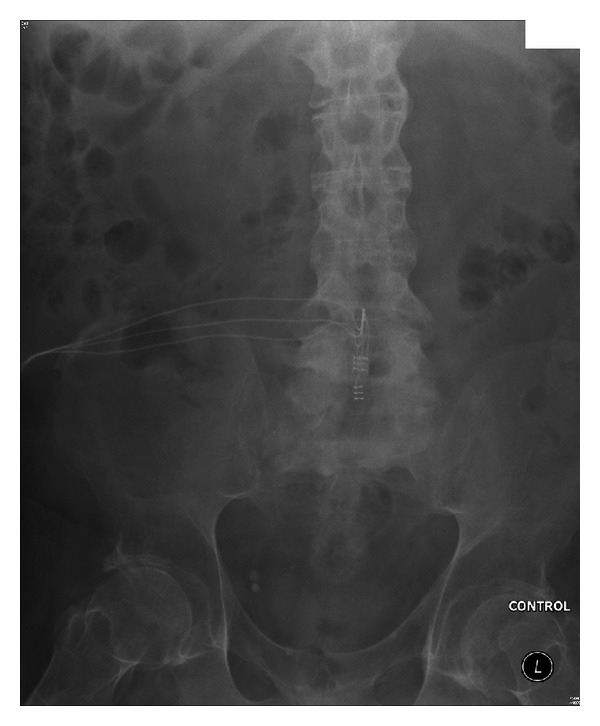
Case 3: X-ray kidneys and urinary bladder, taken in September 2009, showing no radioopaque urinary calculi. The receiver block which had been implanted in the right flank had been removed because of *Staphylococcus aureus* infection.

In 2008, routine intravenous urography revealed some blunting of the right upper pole calyx with some reduction in cortical thickness at the right upper pole compatible with chronic pyelonephritis. Otherwise, both pelvicalyceal systems and ureters appeared normal (Figure 2). Subsequently, this patient developed redness, swelling, and local warmth over the site where the receiver block had been implanted. Microbiology of a wound swab confirmed infection with a heavy growth of *Staphylococcus aureus*, and the receiver block and cables were therefore removed under local anaesthesia (Figure 3). A year later, urine cytology again showed benign epithelial cells and occasional inflammatory cells, with no evidence of neoplasia. Intravenous urography revealed bilateral excretion of contrast without hydronephrosis. In 2012, urine cytology showed predominantly squamous epithelial cells, inflammatory cells with crystals and bacteria in the background but still no evidence of malignancy.

## 3. Discussion

These three cases illustrate what can go wrong with urological implants in spinal cord injury patients. Murphy and associates [3] compared the efficacy and functional durability of the American Medical Systems 800 (AMS 800) artificial urinary sphincter device for patients with neurogenic and nonneurogenic incontinence. The rate of nonmechanical failure was statistically greater in the neurogenic group compared with the nonneurogenic group (*P* < 0.05). Murphy and associates stated that patients who were incontinent as a result of an underlying neurological deficit should be counseled that they might have a higher risk of nonmechanical device failure and a need for reoperation and that their overall long-term continence rates might be poor.

Complications after implantation of sacral root stimulators are known to occur. Kutzenberger [4] reviewed 464 paraplegic patients (220 females and 244 males), who underwent sacral deafferentation and implantation of a sacral anterior root stimulator between 1986 and 2002. Early complications comprised six cases of cerebrospinal fluid leak and five implant infections. Late complications included receiver or cable failures and required surgical repair in 44 patients.

Age-related changes in human anatomy and physiology can also affect the function of urological implants. Clark and associates [5] described the phenomenon of growth spurts associated with lead malfunction that necessitated revision of the implanted sacral nerve stimulators. Four patients underwent sacral nerve modulation at an average age of 12.1 years. Although these patients reported initial success, three patients subsequently required a total of five revisions due to lead malfunction with an average of 1.5 years between surgeries. In those requiring revision, the average somatic growth between revisions was 8.1 cm. Somatic growth is therefore a possible cause of lead malfunction necessitating revision of the sacral nerve stimulator.

One of our patients developed penile retraction and was unable to wear a penile sheath. This case highlights the fact that some spinal cord injury patients may not be able to maintain a condom catheter securely due to inadequate penile shaft length and disappearance of the corpora beneath the pubic fat pad when in the sitting position. Surgical management of this condition includes (i) lysis of the suspensory ligament of the penis; (ii) approximation of the infrapubic soft tissues posterior to the corpora so as to advance the corpora anteriorly and buttress against posterior migration (retraction) in the sitting position; (iii) maximizing the length and fit of prosthetic rods; and (iv) when indicated, release of infrapubic skin tethering with a modified Z-plasty technique [6]. Our tetraplegic patient in Case 3 decided instead to use a long-term indwelling urethral catheter. The bladder stimulator in this patient therefore became functionally useless.

The artificial urinary sphincter was first introduced in 1973, and during the next 10 years there were design changes resulting in five different models of the device. The fifth model, the AS 800 (American Medical Systems, Minnetonka, MN, USA) was introduced in 1983 and is still in use today. This has three separate components: a cuff, a pressure regulating balloon, and a pump-control assembly. The components are implanted separately and connected by two tubing connectors. Since 1983, the basic design of the artificial urinary sphincter has been unchanged; however, there have been numerous modifications to the components of the device leading to both increased continence and longer device life. These component changes include narrow back cuffs for bulbous urethral use, smaller (3.5 and 4.0 cm) cuffs, surface-coated cuffs to reduce wear, kink-resistant tubing, tubing sleeves to reduce wear, and sutureless connectors to facilitate making connections and to reduce connector failures. Montague [7] reported that Kaplan-Meier 5-year projections for freedom from any reoperation were 50% for a small series. Complications of artificial urinary sphincter include cuff erosion, infection, urethral atrophy, and mechanical failure. Kim and associates [8] reviewed 124 consecutive index cases of artificial urinary sphincter implantation in men from 1996 to 2006 for complications (infection, erosion, and mechanical failure) with a median followup of 6.8 years. The overall complication rate was 37.0%, with mechanical failure the most common cause (29), followed by erosion (10) and then infection (7). Kim and associates [8] concluded that appreciable complication rates occur for erosion, mechanical failure, and infection in the first 48 months from implantation of the artificial urinary sphincter. Our patient (Case 2) was unable to insert a size 10 French catheter for intermittent catheterisation six months after undergoing implantation of an artificial sphincter, and suprapubic cystostomy was eventually required; his large sacral pressure sore had still not healed completely five years later. 

## 4. Conclusion

Spinal cord physicians should discuss with patients the benefits and risks of minimally invasive alternative procedures to implants (e.g., intermittent catheterisation instead of bladder stimulator) as well as possible delayed complications of implants such as malfunction and infection. Further, spinal cord physicians and patients should be aware of complications such as pressure sores, chest infection, and would infection, to which spinal cord injury patients are more susceptible than able-bodied individuals.
